# A Threat to Quality Cancer Care, Accessibility, and Sustainability: Medicare Payment Policies and Changing Utilization Patterns in Radiation Oncology

**DOI:** 10.7759/cureus.80064

**Published:** 2025-03-04

**Authors:** Kishan M Patel, Tarita Thomas, Dwight E Heron, Constantine A Mantz

**Affiliations:** 1 Department of Radiation Oncology, Northwestern University Feinberg School of Medicine, Chicago, USA; 2 Department of Radiation Oncology, Bon Secours Mercy Health System, Youngstown, USA; 3 Department of Radiation Oncology, GenesisCare US, Fort Meyers, USA

**Keywords:** conversion factor, medicare physician fee schedule, outpatient prospective payment system, radiation oncology, reimbursement, relative value units, relative weights, value-based care

## Abstract

Introduction: Radiation oncology (RO) has undergone dramatic changes in clinical practice and reimbursement policy due to changes in the Medicare payment system, technological innovation in treatment modalities, and changing use patterns. This study aims to analyze the financial impact of these changes between 2016 and 2022 and their implications for the sustainability of practice and patient access.

Methods: We reviewed Medicare beneficiary-level claims data from 2016 and 2022 to determine trends in payment under the Medicare Physician Fee Schedule (MPFS) and Outpatient Prospective Payment System (OPPS). Treatment episodes were classified by modality (external beam radiation therapy (EBRT) and stereotactic body radiation therapy (SBRT)) and cancer type, with financial effects determined by payment policy changes, utilization changes, and conversion factor changes.

Results: The effect of payment policy changes was stable in relative value units (RVUs) and relative weights (RWs) with no combination of cancer type, service type, and practice setting, showing a difference of more than + 8% or -4%. Reimbursement for technical services in free-standing office practices declined by -16.9% for breast cancer and -14.2% for prostate cancer by greater use of hypofractionation. Inflation-adjusted Medicare conversion factors fell 12.2% in hospital outpatient departments and 20.8% in free-standing offices. The aggregate overall effects were favorable due to the geographic adjustment of national payment rates. For breast episodes, the impact sum for technical charges in the hospital outpatient setting was -21.8%, and the overall impact was -15.6%.

Conclusion: RO practices from 2016 to 2022 showed stable RVUs and RWs, indicating minimal changes to payment policy. However, due to transitioning to hypofractionated regimens and lower treatment volumes, reimbursement rates for technical services for breast and prostate cancers significantly declined. Geographic adjustments reduced overall impacts. However, specific policy initiatives are necessary to guarantee fair reimbursement and maintain access to quality cancer treatment.

## Introduction

Radiation oncology (RO) has undergone significant changes in its clinical practices and reimbursement mechanisms over the past few decades, mainly due to the changes in Medicare policies, innovation in treatment modalities, and shifts in use patterns [1-3]. As an integral part of cancer care, RO operates in a complex financial ecosystem where reimbursement policies significantly determine the sustainability of practices, access to care, and the adoption and diffusion of new technologies. Between 2016 and 2022, the evolution of Medicare's payment models, coupled with clinical advancements such as hypofractionation and stereotactic body radiation therapy (SBRT), has changed how care is delivered in the United States, opening a variety of financial pitfalls and opportunities [4-5]. 

The two main reimbursement frameworks at the core of Medicare's payment system are the Medicare Physician Fee Schedule (MPFS) and the Outpatient Prospective Payment System (OPPS) [6]. These frameworks set reimbursement rates for professional and technical services in two distinctly different care settings. The MPFS determines the payment rates by using RVUs with a national conversion factor, a dollar amount applied to convert RVUs to payment amounts. RVUs have three components: work RVU, representing the physician's effort and expertise in furnishing a service; practice expense RVU, representing operating expenses such as equipment and staff; and malpractice RVU, representing costs associated with liability. The total compensation is calculated using the formula: Compensation = (Work RVU + Practice Expense RVU + Malpractice RVU) × Conversion Factor [6]. 

The Omnibus Budget Reconciliation Act of 1989 introduced budget neutrality in Medicare part B payment RVUs. The requirement states that changes in RVUs cannot increase Medicare Part B payment spending by more than $20 million in a year [6]. This principle of budget neutrality has been a legislative mandate where the increase in payment for some services must be offset by the reduction in others to maintain the overall budget at a fixed level. To achieve neutrality, the Center for Medicare & Medicare Services (CMS) uses a conversion factor. This has exerted continuous downward pressure on the conversion factor and, disproportionately, on high-cost specialties like RO, which demand heavy infrastructure and equipment investments [4]. With this high fixed-cost structure, practices are especially vulnerable to reimbursement cuts [7]. 

The OPPS, which determines payments for hospital outpatient services, encourages efficiency by using geographic adjustments and bundled payment models [8]. Those systems can disadvantage freestanding centers and rural practices that do not have the patient volumes or resources to adapt to fluctuations in revenue [9]. 

Hypofractionation - the delivery of fewer treatment sessions at higher doses per fraction - is a powerful example of the financial complexities of these policy frameworks. While hypofractionation offers both clinical benefits and greater convenience for patients in selected clinical scenarios, it also reduces the number of reimbursable treatment sessions, which in turn results in significant revenue declines for practices that rely on technical reimbursements under the MPFS [10]. Hospital outpatient departments, reimbursed under the OPPS, often do somewhat better because of geographic payment adjustments and more significant patient volumes [11]. 

SBRT has helped offset some of these declines in the adoption rate. With greater technical complexity and relative work value, SBRT commands higher reimbursement rates for specific cancer treatments, such as metastatic disease [12-13]. That said, it also serves to drive a wedge between resource-rich practices that can adopt such advanced technologies and those with more limited resources [14].

Geographic inequalities further compound these inequalities. Urban health practices benefit from changes that allow for higher operational costs, while rural providers face financial vulnerabilities that put access to medical care at risk [15]. These disparities and policy-driven changes in the utilization patterns underline the need for holistic strategies to ensure fair access and long-term sustainability [16].

This study examines the financial implications of clinical innovation, payment policy, and utilization trends in RO from 2016 through 2022. We evaluated the interrelated factors to highlight the challenges and opportunities of payment policies and potential reforms.

## Materials and methods

This is a descriptive retrospective cohort study of RO services furnished to all Medicare beneficiaries treated in the facility setting during a recent historical period. This study follows the Strengthening the Reporting of Observational Studies in Epidemiology (STROBE) reporting guideline [17]. 

Data sources 

This study used Medicare beneficiary-level, final action claims data abstracted from the following Limited Data Set (LDS) Standard Analytic Files (SAF) for each calendar year, 2016 through 2022: Outpatient Revenue Center Files, Outpatient Base Claims Files, Carrier Line Files, and Carrier Base Claim Files [18]. Medicare Part B technical services furnished in either the hospital outpatient or off-campus provider-based department setting are contained within the Outpatient Files. Part B technical services delivered in the freestanding office and professional services provided by physicians in either hospital department or office setting are contained within the Carrier Files. Medicare beneficiaries whose benefits were administered through Part C, or Medicare Advantage health plans, were identified through the 2016-2022 Master Beneficiary Summary Files and excluded from this analysis, as reimbursement information for their services is not available through the SAFs. 

Analysis file preparation 

Individual claims reporting radiation therapy (RT) services were extracted from the SAFs and assigned to 90-day care episodes following the method described in the RO-APM Final Rule and detailed further here [19]. 

Procedure codes specific to RT professional, technical, and global services were used to isolate pertinent claims for this analysis from the Outpatient Revenue Center and Carrier Line Files. These files include provider payment data and dates of service in addition to procedure codes. 

The isolated claims were then appended with diagnosis codes obtained from the Outpatient Base and Carrier Base Files, which are linked to individual claims through Beneficiary ID and Claim Number data fields common to all SAFs. Therefore, all abstracted claims included procedural, payment, and diagnosis information for each Medicare beneficiary who received RT services between 2016 and 2022. Claims for individual beneficiaries were then organized chronologically according to dates of service. 

Day 1 of an episode was determined as the date of service for which a Clinical Treatment Planning service (i.e., HCPCS 77261, 77262, or 77263) was reported. All subsequent RT services appearing in the claims record were then assigned to the same episode through day 90. A new episode for the same beneficiary was designated if another Clinical Treatment Planning service was reported more than 120 days from the start of the immediately prior episode. Treatment modality for an episode was assigned according to the mix of Treatment Delivery procedure codes (e.g., EBRT alone, EBRT plus Brachy, etc.) reported during the episode. Diagnosis for an episode was assigned according to the plurality of ICD 10 codes reported on episode claims. 

Episodes utilizing either external beam radiation therapy (EBRT) or stereotactic therapy (Stereo) were retained for this analysis, and all other modality episodes were excluded. EBRT and Stereo episodes together comprised 88.7% of episodes over the study period and have observed significant intramodality (e.g., increased use of EBRT hypofractionation for prostate and breast cancers) and intermodality (e.g., decreased use of EBRT and increased use of Stereo for metastases) utilization shifts with commensurate impacts on allowed charges over the period. Any episode, regardless of modality, was excluded if care were delivered in (1) cancer hospitals exempted from Medicare’s Prospective Payment System (PPS); (2) Critical Access Hospitals (CAHs), which are reimbursed under a modified PPS methodology; and (3) Maryland and Vermont hospitals participating in a Medicare Alternative Payment Models. Finally, any episode starting after October 4, 2022, was excluded to allow for a 90-day run-out period necessary for episode completion. 

Determination of payment policy, utilization, and conversion factor change impacts 

In the context of this study, payment policy change refers to the variation over time of (1) relative value units (RVUs) for those RT services reimbursed under the Medicare Physician Fee Schedule (MPFS), which include technical services performed in the office setting and professional services performed in either the office or hospital setting, and (2) relative weights (RW) for those RT services reimbursed under the Outpatient Prospective Payment System (OPPS), which include technical services performed in the hospital setting. While conversion factor updates are also an element of payment policy, conversion factor impact is evaluated separately in this analysis as its effect applies equally across all services in a fee schedule, whereas RVU and RW effects are more specialty-specific. Both RVU and RW represent the relative value of providing a given healthcare service compared to other services under their respective payment systems based largely on the expense and time necessary in the provision of that service. Under the MPFS, CMS may affect the RVU of a service by updating the costs of that service’s equipment and supplies, adopting new utilization assumptions for expensive equipment such as linear accelerators, and myriad other adjustments that may be applied to the complex calculation that determines service RVU. Under the OPPS, CMS may likewise affect the RW of a service by updated hospital cost data, introduction of new medical procedures with subsequent RW adjustment of existing procedures to meet statutory requirements of budget neutrality, among many other factors. Once calculated for a calendar year (CY), RVU and RW are multiplied by conversion factors to establish national payment rates for their respective services. 

To estimate the impact of changes in Medicare payment policy upon RVU and RW for RT services between 2016 and 2022 and to exclude the impact of changes in technology utilization and conversion factor during the same period, episodes performed during CY 2022 were selected and isolated from remaining episodes. Service units for each procedure code paid by Medicare that year were summed and divided by the total number of episodes to determine the average number of units per procedure per episode. These values were then each multiplied by the RVU and RW for their corresponding procedure codes for CY 2016 and CY 2022, respectively, and the resulting RVU and RW were summed to calculate the average number of RVU and RW per episode for CY 2016 and CY 2022. Therefore, any differences in relative values between these years represent the effect of changes in payment policy as utilization is held constant through the use of a single calendar year’s episodes. 

Utilization change refers to the variation over time in the prescription of fraction number for EBRT cases and the distribution of EBRT-to-Stereo cases among episodes. Examples of utilization change as defined here include increased share of hypofractionated treatment courses for breast cancer and greater adoption of stereotactic therapies for oligometastatic disease over a recent historical period. While it may be readily appreciated that decreasing EBRT fraction number will reduce allowable charges on a per-case basis, the net financial effect of converting EBRT cases to Stereo cases is more difficult to grasp as unit pricing of treatment delivery services and the prescribing of services ancillary to treatment delivery may vary greatly between EBRT and Stereo cases. To estimate the impact of utilization changes between 2016 and 2022 while excluding the influence of changes in payment policy and the conversion factor, episodes performed during CY 2016 and CY 2022 were isolated for analysis. Service units for each Medicare procedure code were summed and divided by the total number of episodes treated in each respective year, yielding the average number of units per procedure per episode per calendar year. These averages were then multiplied by the RVU and RW associated with their respective procedure codes for CY 2022. The resulting RVU and RW values were summed up to calculate the average RVU and RW per episode for both CY 2016 and CY 2022. This method ensures that differences in relative values between the two years reflect only the effect of utilization changes, as payment policy factors were held constant by using a single calendar year’s relative values. 

Conversion factor change is the difference between the 2016 and 2022 conversion factors under the MPFS and OPPS fee schedules, adjusted for inflation. The inflation index used for this adjustment is the Consumer Price Index for All Urban Consumers (CPI-U), which measures price change over time for a representative basket of goods and services, and the base year used is CY 2024. 

## Results

A total of 135,296 hospital outpatient episodes and 57,720 freestanding office episodes utilizing either EBRT or Stereo modalities were initiated and completed during CY 2022. The distribution of cancer types among these episodes was 18.1% breast, 18.0% prostate, 19.8% secondary sites, and 44.1% other primary sites. For CY 2016, 135,484 hospital outpatient and 69,720 freestanding office EBRT or Stereo episodes were initiated and completed with the following cancer type distribution: 16.8% breast, 14.7% prostate, 19.2% secondary sites, and 49.2% other primary sites. 

The effect of payment policy change upon RO service RVUs and RWs between 2016 and 2022 is provided in Table 1.

**Table 1:** Effect of Medicare payment policy on relative values, CY 2022 versus CY 2016 (service utilization held constant at CY 2022 values)

	HOSPITAL Technical (relative weights)		
	2022 policy	2016 policy	% Diff
Breast	114.5	108.6	5.4%
Prostate	218.7	223.9	-2.3%
Secondary site	109.1	109.6	-0.5%
Other primary site	151.3	152.8	-1.0%
	OFFICE Technical (RVUs)		
	2022 policy	2016 policy	% Diff
Breast	243.2	245.9	-1.1%
Prostate	500.3	463.4	7.9%
Secondary site	179.7	186.8	-3.8%
Other primary site	292.4	285.6	2.4%
	ALL Professional (RVUs)		
	2022 policy	2016 policy	% Diff
Breast	63.9	61.2	4.4%
Prostate	108.4	102.9	5.4%
Secondary site	55.4	53.0	4.4%
Other primary site	69.5	66.2	5.0%

Relative values for all professional and technical services were stable throughout the period with no combination of cancer type, service type (professional or technical), and practice setting observing a difference of greater than +8% or less than -4%. 

The impact of utilization changes upon RO service RVUs and RWs during the study period is provided in Table 2.

**Table 2:** Effect of service utilization on relative values, CY 2022 versus CY 2016 (Medicare payment policy held constant at CY 2022 values)

	HOSPITAL Technical (relative weights)		
	2022 utilization	2016 utilization	% Diff
Breast	114.5	134.7	-15.0%
Prostate	218.7	261.8	-16.5%
Secondary site	109.1	97.5	11.9%
Other primary site	151.3	145.0	4.3%
	OFFICE Technical (RVUs)		
	2022 utilization	2016 utilization	% Diff
Breast	243.2	292.6	-16.9%
Prostate	500.3	583.3	-14.2%
Secondary site	179.7	167.2	7.4%
Other primary site	292.4	307.8	-5.0%
	ALL Professional (RVUs)		
	2022 utilization	2016 utilization	% Diff
Breast	63.9	72.0	-11.3%
Prostate	108.4	123.6	-12.3%
Secondary site	55.4	52.8	4.9%
Other primary site	69.5	73.6	-5.5%

**Figure 1:**
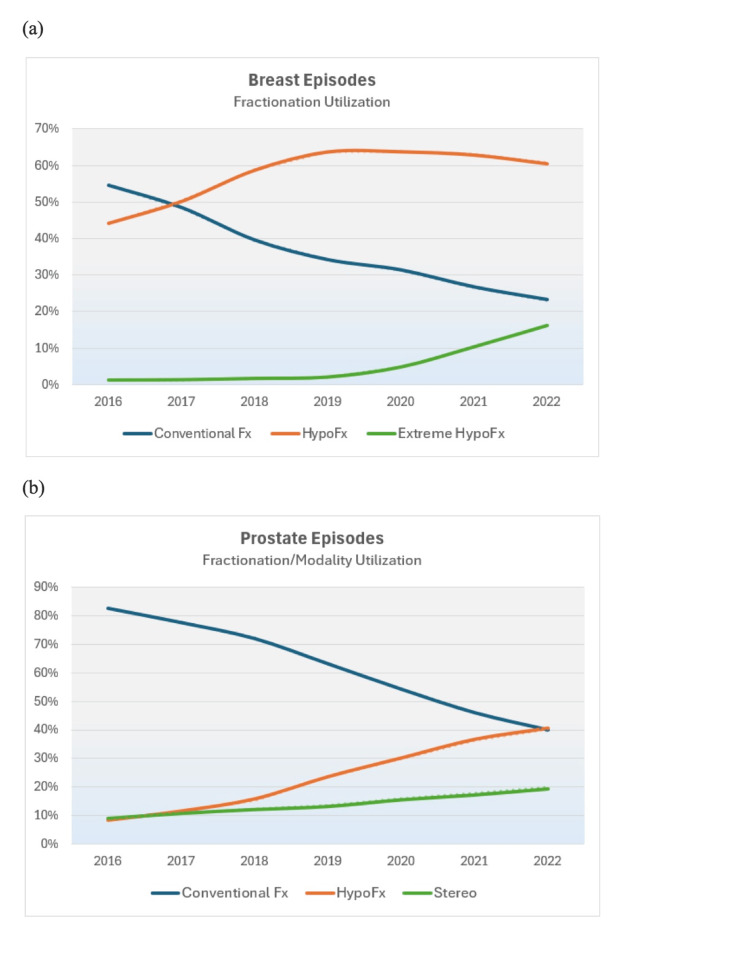
Fractionation schedule and stereotactic therapy utilization for (a) breast and (b) prostate episodes, 2016-2022 Extreme HypoFx indicates breast episodes of five fractions.

Breast and prostate episodes observed relative value declines of -14.2% to -16.9% for technical services in either practice setting and declines of -11.3% to -12.3% for professional services, largely contributed to by increased hypofractionation for breast episodes and increased hypofractionation and stereotactic therapy for prostate episodes. For breast episodes, loss of treatment delivery technical relative values accounted for 77.7% of total technical losses in the hospital outpatient setting and 85.6% of total technical losses in the freestanding office setting (data not shown). For prostate episodes, loss of treatment delivery technical relative values accounted for 87.6% of total technical losses in the hospital outpatient setting and 71.2% of total technical losses in the freestanding office setting (data not shown). Regarding professional services, loss of weekly physician treatment management services accounted for 86.9% and 46.9% of total professional relative losses for breast and prostate episodes, respectively (data not shown). Trends in the adoption of hypofractionated and stereotactic treatment schedules for breast and prostate cancer are illustrated in Figure 1.

Secondary site episodes observed net increases in relative values for all service types and practice settings, largely on the basis of greater utilization of better-reimbursing stereotactic treatment and lesser use of short-course EBRT (data not shown). 

The impact of Medicare conversion factor updates under OPPS and MPFS is provided in Table 3.

**Table 3:** Effect of Medicare conversion factor updates, CY 2022 versus CY 2016, adjusted for inflation

	Conversion Factor (CPI adjusted (2024 $))		
	2022	2016	% Diff
Hospital	$89.77	$102.26	-12.2%
Office	$36.91	$46.59	-20.8%

When adjusted for inflation, Medicare conversion factors have decreased by -12.2% in the hospital outpatient setting and by -20.8% in the freestanding office setting and for physician professional payments between 2016 and 2022. These factors convert RWs and RVUs into allowable Medicare charges for facilities and providers. 

The aggregate effect of changes in Medicare payment policy, service utilization, and Medicare conversion factors is provided in Table 4.

**Table 4:** Aggregate effect of Medicare payment policy, service utilization, and Medicare conversion factor updates upon average episode Medicare allowable charges, CY 2022 versus CY 2016

	HOSPITAL Technical (2024 $)		
	2022	2016	% Diff
Breast	$10,575	$12,534	-15.6%
Prostate	$20,444	$26,509	-22.9%
Secondary site	$9,916	$9,231	7.4%
Other primary site	$13,609	$14,121	-3.6%
	OFFICE Technical (2024 $)		
	2022	2016	% Diff
Breast	$8,892	$13,301	-33.2%
Prostate	$18,850	$24,344	-22.6%
Secondary site	$7,041	$7,929	-11.2%
Other primary site	$10,596	$13,104	-19.1%
	ALL Professional (2024 $)		
	2022	2016	% Diff
Breast	$2,276	$3,078	-26.0%
Prostate	$3,986	$5,202	-23.4%
Secondary site	$1,956	$2,150	-9.0%
Other primary site	$2,475	$3,013	-17.9%

These values represent average Medicare allowable technical and professional charges for breast, prostate, secondary site, and other primary site episodes treated in 2022 and 2016 in 2024 dollars. With the exception of secondary site episodes treated in the hospital outpatient setting, losses are observed for every other combination of cancer type, service type, and practice setting over the period, with the greatest losses on a percentage basis observed for office-based technical services. Note that the percentage values in Table 4 are slightly more favorable than the corresponding mathematical sums of percentage impacts of payment policy, utilization, and conversion factor updates. For example, the percentage impact sum for technical charges for breast episodes in the hospital outpatient setting is -21.8% ([+5.4%] + [-15.0%] + [-12.2%]), whereas the actual overall impact is -15.6%. This favorable difference is explained by the geographic adjustment of national payment rates that occurs prior to disbursement to individual providers and reflects the distribution of radiation therapy departments and offices in markets where local costs generally exceed national averages. 

## Discussion

The findings of this study demonstrate the intricate interplay between Medicare reimbursement policies, shifts in utilization patterns, and the corresponding financial repercussions for RO practices from 2016 to 2022. Throughout this period, the relative stability of RVUs and RWs for RO services, varying only between +8% and -4%, indicates minimal changes to the payment policies themselves [4,6]. Despite this stability, there was a significant decline in reimbursement for technical services, particularly within the context of treatment episodes for breast and prostate cancers. These reductions were primarily driven by the transition to hypofractionated treatment regimens, which, while clinically effective, result in fewer billable fractions and considerable financial implications, especially for independent office settings [8,10]. In addition, the relative value of professional services experienced a decline, particularly in the realm of weekly management services, further exacerbating the financial challenges associated with breast and prostate cancer cases [3,9]. 

Trends in RO during this period have notably influenced reimbursement models. The rise of hypofractionation, particularly in the treatment of breast and prostate cancers, has contributed to a decrease in overall reimbursement due to lower treatment volumes [9,15]. Conversely, the growing adoption of stereotactic treatment for oligometastatic cancer cases has counterbalanced these declines by capitalizing on the higher relative value associated with complex treatment modalities [12,18]. In addition, shifts in the relative distribution of cancer types, characterized by a modest decline in breast and prostate cases alongside an increase in secondary site cases, have further impacted overall reimbursement trends. For example, episodes of breast cancer treatment experienced significant declines in technical value, with reductions of -16.9% in independent offices and -15.0% in hospital outpatient settings, attributed mainly to a drop in fractionation volumes [1,13]. In contrast, episodes related to secondary site cancers have benefited from a rising utilization of stereotactic therapy, with their relative value increasing by 11.9% in hospital outpatient settings alone [14]. 

Geographic adjustments have been pivotal in mitigating reimbursement shortfalls for certain RO practices. Those in high-cost urban areas have benefited more from these adjustments than rural practices, where reimbursement rates may fall short of covering operational expenses [6-7]. This disparity poses significant implications for healthcare access, as rural health facilities face heightened financial instability, potentially limiting access to RO services in regions that already lack adequate health resources. For example, the overall declines in reimbursement for breast cancer care episodes were less severe than anticipated due to these geographic cost adjustments, highlighting the crucial role such mechanisms play in cushioning the impact of broader policy changes [1,20]. 

The challenges faced by RO are not unique; other interventional specialties have also encountered declines in reimbursement [8]. Interventional Radiology and Vascular Surgery have addressed these challenges by optimizing care pathways, adopting minimally invasive techniques, and transitioning procedures to outpatient settings to better align with value-based care models [11,21-22]. These strategies offer valuable insights for RO as they adapt to the financial realities of hypofractionation and stereotactic therapy. By embracing value-based care frameworks prioritizing outcomes over volume, RO can enhance clinical outcomes and financial sustainability, ensuring continued access to high-quality care for Medicare beneficiaries [1-2,16]. 

Limitations

Despite the strengths intrinsic to our study, we recognize several limitations. Our analysis, based on a large national dataset derived from Medicare claims data, is quite robust; nevertheless, the possibility of unmeasured confounding variables that might affect the finding remains. For example, socioeconomic factors related to patient characteristics, such as income, supplementary insurance status, and availability of care for unforeseen geographic confounding variables, were not specifically included in our dataset. In addition, practice style variations based on geographic location and clinician and institutional preferences for specific fractionation regimens might belie reimbursement trends that may not be fully accounted for in our analytical models.

The impact of geographic differences on reimbursement determinations is also another possible confounding factor. While our study employs standardized national payment rates, reimbursement is most frequently determined by geographic practice cost indices (GPCIs), which account for regional differences in labor costs, operational costs, and malpractice insurance rates. Future analyses incorporating these geographic adjustments could provide better insight into how these variations between regions affect reimbursement within RO. 

## Conclusions

The evidence provided in this report has established a temporal correlation between changes in reimbursement and observed behavior in clinical practice. However, it is important to highlight that the findings may not imply a causal relationship. Instead, while changes in reimbursement could have an effect on clinical decision-making, causality is often a substantial challenge to determine, given the complex interactions between patterns of clinical practice, physician incentives, and patient preferences. Therefore, additional studies, including prospective research and qualitative evaluation, are required to fully understand the broader implications of these trends on patient outcomes and access to care. This research seeks to stimulate a more comprehensive and open discussion regarding the changing reimbursement landscape in RO.
